# A novel choline chloride/graphene composite as a shale inhibitor for drilling fluid and the interaction mechanism

**DOI:** 10.1039/d2ra05085d

**Published:** 2022-10-24

**Authors:** Heming Zhu, Daqi Li, Xiangyang Zhao, Shaocong Pang, Yuxiu An

**Affiliations:** Key Laboratory of Shale Oil and Gas Enrichment Mechanism and Development, Sinopec Research Institute of Petroleum Engineering Changping District Beijing 100101 China zhuhm.sripe@sinopec.com lidq.sripe@sinopec.com zhaoxy.sripe@sinopec.com; School of Engineering and Technology, China University of Geosciences (Beijing) Haidian District Beijing 100083 China P7426cong@163.com anyx@cugb.edu.cn; Key Laboratory of Deep Geo Drilling Technology, Ministry of Land and Resources Beijing 100083 China

## Abstract

For wellbore stability in shale formations, the development of environmentally friendly and efficient shale inhibitors is urgently needed. Herein, we report the preparation of choline chloride-modified graphene (Ch-G). The inhibition and interaction mechanisms of choline chloride-modified graphene on montmorillonite were also investigated. We evaluated the inhibition of Ch-G *via* linear swelling and rolling recovery and selected the inorganic salt inhibitor KCl as the control group. The lowest swelling height of 2.10 mm and the highest rolling recovery of 78.87% were achieved, indicating the excellent inhibition performance of Ch-G. The mechanism of inhibition of Ch-G was determined by Fourier transform infrared spectroscopy, X-ray diffraction, X-ray photoelectron spectroscopy, transmission electron microscopy, scanning electron microscopy, and atomic force microscopy. The Ch-G formed hydrogen bonds, coordination, and electrostatic interactions with the surface of montmorillonite and entered the montmorillonite *via* intercalation to achieve the inhibition. The increase in the nitrogen atom content in Ch-G led to the production of more positive ions and the formation of more ion bands, which enhanced the ability to inhibit shale hydration. The addition of Ch-G produced larger montmorillonite sheets, demonstrating its effective inhibition ability, which is needed to enable drilling fluids to stably drill into shale formations.

## Abstract 2

The chemical structure of the choline chloride/graphene oxide composite.

## Graphical abstract

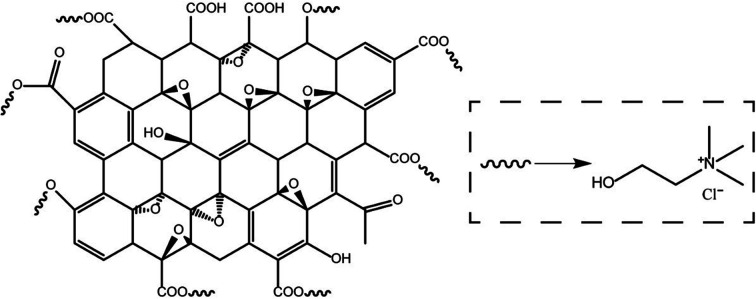

## 1. Introduction

Oil and gas resources are an indispensable part, and there is an urgent need for efficient drilling to extract oil and gas resources.^1^ Shale is used as source and reservoir rock, and 75% of drilling formations for oil and gas resources are in shale formation.^2^ As the main component of shale, clay minerals, mainly composed of montmorillonite, will expand when they encounter water, causing wellbore safety problems.^3,4^ Although clay minerals in shale are not hydrated in oil-based drilling fluids (OBDFs), OBDFs have problems such as high cost, non-biodegradability, and being harmful to environmental health.^5^ Nowadays, relevant environmental protection agencies are becoming more and more strict with treatments that are harmful to the environment, and finally water-based drilling fluids (WBDFs) have been selected for drilling. However, drilling with WBDFs in shale formations to exploit resources faces a major problem, that is, the shale is extremely easy to swelling. This means that it will bring wellbore stability problems such as hole collapse and economic losses and will seriously affect the progress of the project.^6^ Therefore, inhibiting the hydration of shale is the key for WBDFs to be used for drilling in shale formations.

An inhibitor is a treatment agent that inhibits the hydration of shale and is added to form an inhibitory drilling fluid.^7^ Much research has been done on shale inhibitors; the earliest used was the inorganic salt KCl, which switched weakly hydrated potassium ions (K^+^) for sodium ions (Na^+^).^8^ Other inhibitors include CaCl_2_, NH_4_Cl, modified hard bitumen, and bitumen.^9,10^ KCl/sodium silicate drilling fluid can inhibit shale hydration in the formation with strong sensitivity.^11^ However, high concentrations of K^+^ have not been allowed to exist in the formation due to consideration of environmental protection.^12–15^ In addition, functional cationic polyquaternary amine polymers, amphoteric poly amino acids, nonionic polymers, and cationic polymers have also been studied.^16–18^ Quaternary ammonium groups were introduced to synthesize inhibitors together with gelatin, which showed better inhibition at high temperatures.^19^ Xu *et al.* synthesized polymer microsphere emulsions, which could block shale nanopores and reduce shale hydration capacity due to their tiny particle size.^20^ Most of the inhibitors played the role of inhibiting shale hydration by entering the montmorillonite intercalation, charge neutralization, and plugging shale pores. Changes in the wettability of shale surfaces by inhibitors were also investigated.^21^ Organosilicon quaternary ammonium salts were prepared to act as shale inhibitors, which could form hydrophobic films on shale surfaces through chemical reactions. Therefore, the wettability of the shale surface changed, resulting in a greatly reduced water adsorption capacity. In strengthening the hydrophobic shielding of clay, imidazolyl-based ionic liquids for shale inhibitors have also been studied.^22,23^ However, some shale inhibitors still have low toxicity and poor inhibitory performance at high temperatures, even having a bad impact on drilling fluid rheology.^24^ It is particularly urgent to research an environmentally friendly and effective shale inhibitor.^19,25,26^

As a research hotspot in this century, graphene materials have attracted the interest of researchers because of their good physical and electronic properties.^27–29^ In the field of drilling engineering, graphene has been studied as a filter loss agent, and it has been found to cause a good filter loss reduction.^30^ In view of the lubricating properties of graphene materials, lubricants based on graphene materials were used in water-based drilling fluids.^31^ They relied on adsorption to form a layer of film on the surface of the filter cake, which reduced the wear of drilling tools and slightly reduced the filtration of drilling fluid. Recently, there have been studies on graphene materials for shale inhibitors.^32^ Lv *et al.* obtained a shale inhibitor with better performance by modifying dodecylamine and graphene oxide.^33^ Since graphene is a nanosheet with an atom-thick 2-position conjugated structure, it can form a dense film on shale and it can also block the nanopores of shale. Choline chloride could also be used in shale inhibitors due to its stable anti-swelling properties and high efficiency, as well as its non-toxic and biodegradable advantages.^34^ It is well known that there are many nanopores in shale. Graphene is a flexible nanosheet that can block the nanopores of shale formations, preventing clay swelling caused by water intrusion.^35^ Therefore, graphene materials and choline chloride materials are considered to have great potential for environmentally friendly shale inhibitors.

In this paper, choline chloride-modified graphene (Ch-G) was obtained by blending graphene and choline chloride. The linear swelling height and rolling recovery rate were tested to evaluate the performance of Ch-G in inhibiting hydration swelling of shale in water-based drilling fluids. The inhibition mechanism of Ch-G was also investigated by Fourier transform infrared spectroscopy, X-ray diffraction, X-ray photoelectron spectroscopy, scanning electron microscopy, transmission electron microscopy, and atomic force microscopy.

## 2. Experimental section

### 2.1 Materials

**Fig. 1:**
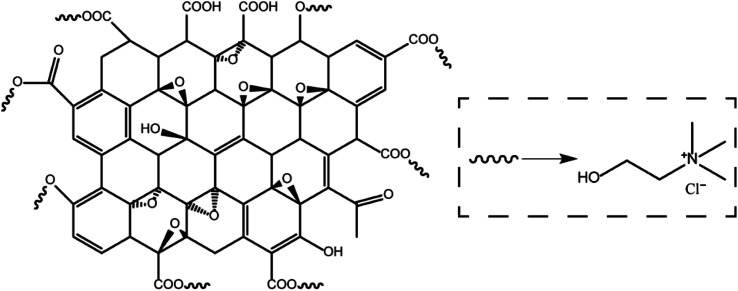
The chemical structure of the choline chloride/graphene oxide composite.

Montmorillonite (MMT) was provided by HuaWei Company. Choline chloride was obtained from Alfa Reagent Company. Graphene oxide (GO) was provided by XianfenNano Company. The other materials such as KCl, xanthan gum (XC) were all provided by a domestic reagent company. All materials were used as obtained without further purification. The chemical structure of the choline chloride/graphene oxide composite is shown in Fig. 1.

### 2.2 Methods

#### 2.2.1. Preparation of Ch-G

For the synthesis of Ch-G, refer to a previous report by other researchers.^36,37^ Graphene oxide was added to water in a mass ratio of 0.15 : 200 and dissolved to uniformity *via* mild sonication. Then, 40 ml of choline chloride was added to the solution and refluxed at 80 °C for 8 hours.

#### 2.2.2. Linear swelling tests

MMT (5 g) was poured into the pressure tank and pressed at 10 Mpa for 5 min to prepare a sample. The MMT sample was placed in the CPZ-2 dual channel linear dilatometer, then the water, 4 wt% KCl, 0.2 wt% GO and 4 wt% Ch-G inhibitor solutions were added, respectively. The value was recorded as zero when the inhibitor solutions were poured in. The expansion heights of MMT in water, KCl and Ch-G solutions were determined with time.

#### 2.2.3. Rolling recovery tests

Here, 20 g of shale debris with 6–10 mesh, with weight denoted as *W*_1_, was added to 300 ml of water and different inhibitor solutions (0.3 wt% XC, 4 wt% KCl, 0.2 wt% GO and 4 wt% Ch-G solutions), and aged at 120 °C for 16 h in a high-temperature roller furnace XGRL-4A. After cooling to room temperature, the resulting shale was thoroughly dried and sieved to 40 mesh and the weight denoted as *W*_2_. The rolling recovery was calculated using the following formula: rolling recovery = *W*_2_/*W*_1_.

### 2.3 Characterization techniques

#### 2.3.1. FT-IR measurements

A Nicolet iS50 spectrometer was used for FT-IR analysis of MMT and Ch-G/MMT.

#### 2.3.2. XRD measurements

A D8 Advance diffractometer was used to perform MMT and Ch-G/MMT analysis. The pattern was collected with 2*θ* angle scanning between 5° and 10°. The basal interlayer spacing *d*_(001)_ was determined using Bragg's equation: 2*d* sin *θ* = *nλ* (*λ* = 0.15406 nm, *n* = 1).

#### 2.3.3. XPS measurements

An ESCALAB 250 was used for XPS analysis of GO, Ch-G, MMT/GO and MMT/Ch-G.

#### 2.3.4. SEM

Here, 0.001 wt% GO and Ch-G solutions were prepared, and their microstructures were observed using a Quanta 200F instrument.

#### 2.3.5. TEM

The analyses of pristine MMT and MMT in 0.001 wt% Ch-G solutions were performed using an F20 transmission electron microscope.

#### 2.3.6. AFM

An SPM-9600 instrument was used to obtain the images of pristine MMT and 0.001 wt% Ch-G/MMT hybrids.

## 3. Results and discussion

### 3.1 FT-IR

**Fig. 2:**
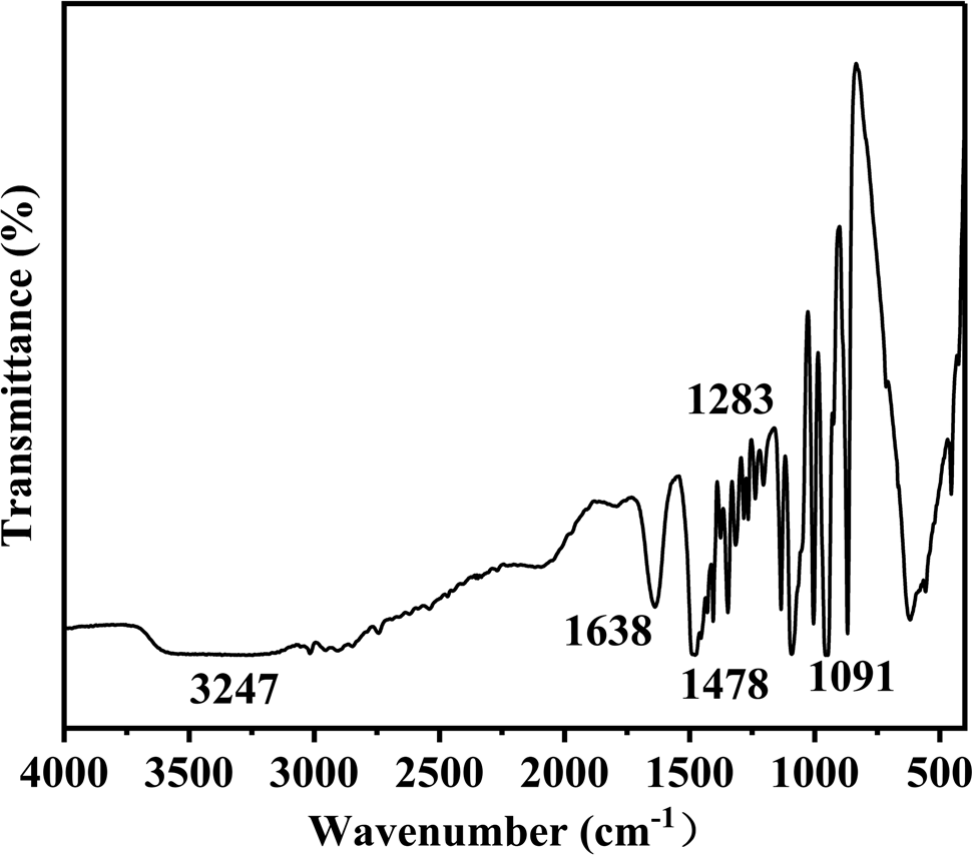
The FT-IR spectrum of Ch-G.

The structure of Ch-G was characterized by FT-IR and the spectrum is shown in Fig. 2. Peaks at 1091 cm^−1^ and 1283 cm^−1^ were attributed to the bending vibration absorption peaks of C–O and C–O–C, respectively. The peak at 1638 cm^−1^ was due to the C=C skeleton vibration of the benzene-like ring on graphene oxide. The curved vibration peak of –CH_2_ on the chlorine chloride appeared at 1478 cm^−1^. The peak at 3247 cm^−1^ was due to the stretching vibration of –OH. All these proved the successful preparation of Ch-G.

### 3.2 Linear swelling tests

**Fig. 3:**
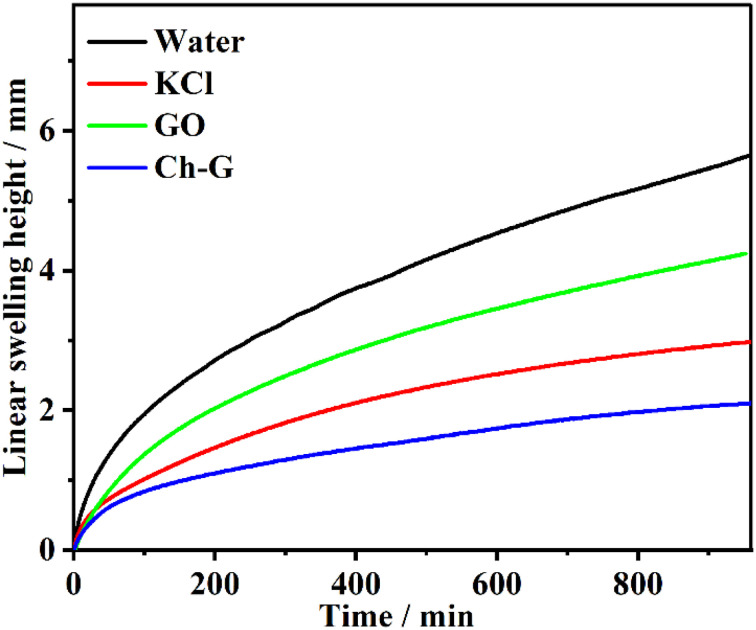
The changes in the linear swelling height of MMT in different inhibitor solutions with time.

The inhibition effect of the shale inhibitor is reflected in the inhibition of clay hydration and swelling, resulting in a low swelling height. The swelling height curves of compacted bentonite in different inhibitor solutions were obtained by linear swelling tests and can be seen in Fig. 3. The inhibitor solution was divided into a water solution, 4 wt% KCl solution, 4 wt% Ch-G solution and 0.2 wt% GO solutions. The swelling heights of bentonite in these four solutions showed the same trend, increasing rapidly and then slowly. After 400 min, the growth rate of the Ch-G curve gradually stabilized, while the curve of the water solution was in a growing state. The swelling height of bentonite in the Ch-G solution was the lowest, which was 2.10 mm. The swelling height of bentonite in water solution was the highest, and the swelling height reached 5.64 mm after 16 h. The most common inorganic salt KCl inhibitor was selected as the control group. The final swelling height of bentonite in KCl solution reached 2.98 mm; compared with the water solution, the swelling height decreased, and KCl showed an inhibition. Compared with GO, Ch-G reached the reduction rate of the swelling height with 50.47%. This indicated that the introduction of choline chloride played a great role in the inhibitory effect of Ch-G. The results showed that the prepared Ch-G exhibited a good ability to inhibit the hydration and swelling of MMT.

### 3.3 Rolling recovery tests

**Fig. 4:**
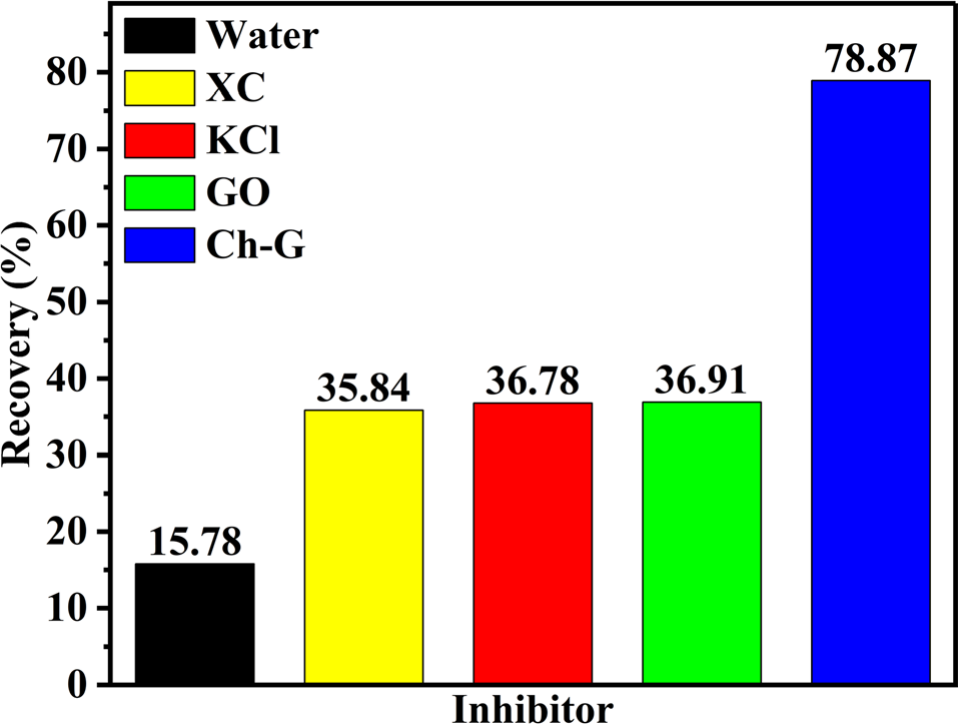
Rolling recovery of different inhibitors.

Shale cuttings are rich in clay and are easily hydrated and dispersed in water. After the inhibition of shale inhibitors, more unhydrated shale debris is recycled in the rolling recovery test. Therefore, rolling recovery tests were performed to evaluate the inhibitory performance of shale cuttings containing unhydrated dispersed clays. Fig. 4 shows the rolling recoveries of shale cuttings in water, XC solution, KCl solution, GO solution, and Ch-G solution at 120 °C. The shale rolling recovery in water was the lowest. Compared with the GO solution, the rolling recovery in the Ch-G solution increased from 36.91% to 78.87%. This indicated that the introduction of choline chloride enabled Ch-G to inhibit the hydration and dispersion of shale cuttings and increase the amount of recoverable shale cuttings. Ch-G could still maintain a high recovery rate after high temperature hot rolling because of the hydrogen bonds and ionic bonds formed between choline chloride and graphene oxide. In actual drilling engineering, a high rolling recovery rate is more conducive to solid-phase separation operations. The high shale rolling recovery rate of Ch-G solution indicated that there was the potential for the application of Ch-G as an effective shale inhibitor.

### 3.4 Inhibition mechanism analysis

#### 3.4.1. FT-IR

**Fig. 5:**
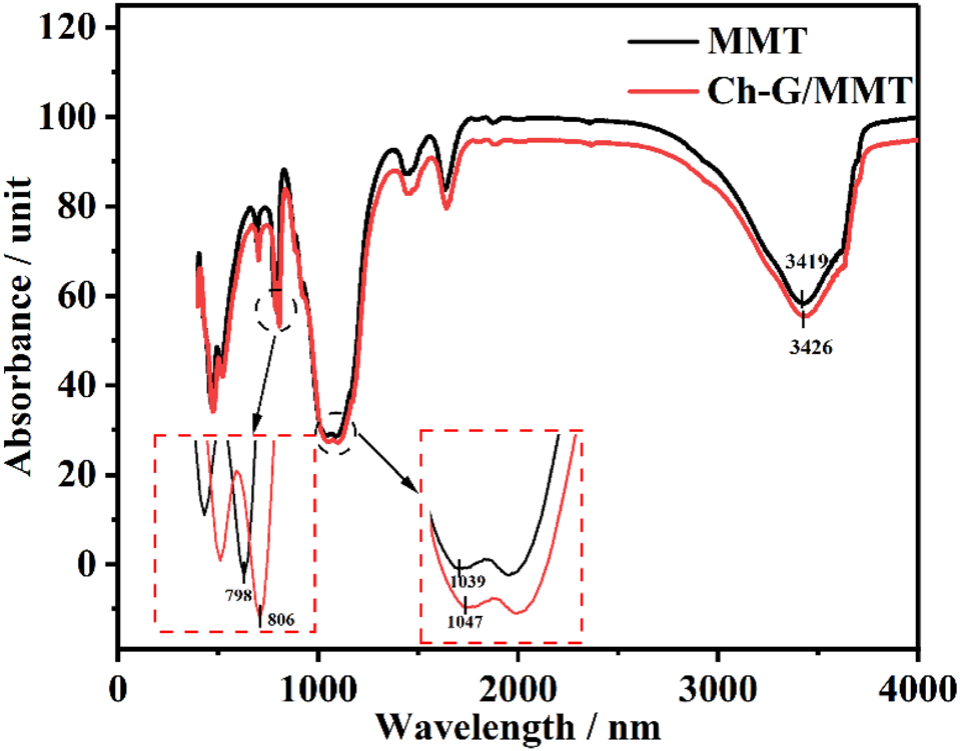
FT-IR spectra of MMT and Ch-G/MMT.

Infrared spectroscopy was used to analyze the interactions between Ch-G and MMT. Fig. 5 shows the FT-IR spectra of MMT and Ch-G/MMT hybrids. Compared with pristine MMT, the peaks for the stretching of O–H shifted from 3419 cm^−1^ to 3426 cm^−1^ in the FT-IR spectrum of Ch-G.^38^ The vibration bands of Al–O and Si–O shifted from 798 cm^−1^ and 1039 cm^−1^ to 806 cm^−1^ and 1047 cm^−1^, respectively. This indicated that there were hydrogen bonds between Ch-G and the MMT surface.

#### 3.4.2. XRD

**Fig. 6:**
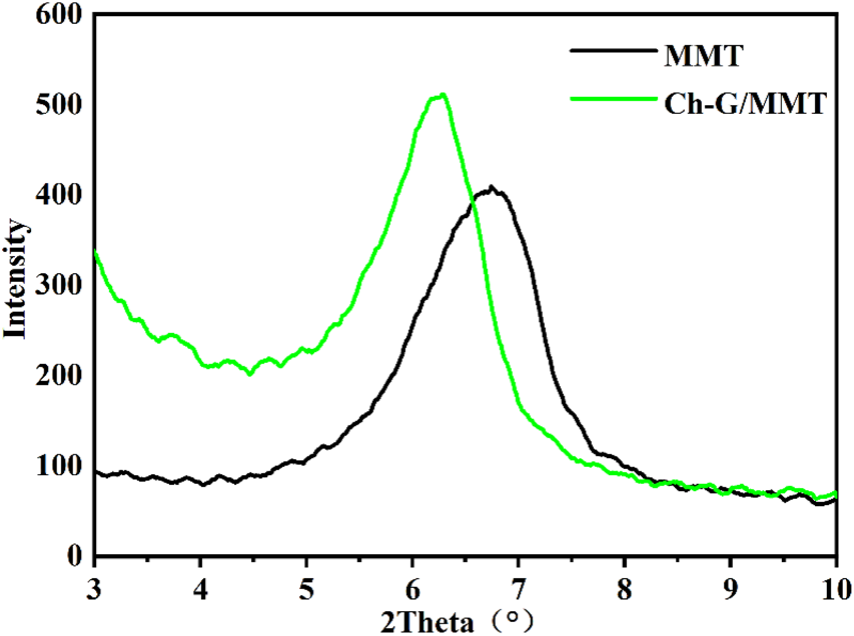
XRD patterns of MMT and Ch-G/MMT.

Fig. 6 shows the *d*_(001)_ of MMT and Ch-G/MMT hybrids. The layer spacing for the pristine MMT was *d* = 1.31 nm; after subtracting the layer thickness and hydrogen bond length, it was found that the MMT swelled. After adding Ch-G, the interlayer spacing changed from *d* = 1.31 nm to *d* = 1.41 nm, demonstrating that Ch-G was inserted into the MMT interlayer. Hydrogen bonds were formed between N atoms, –OH and O atoms on the interlayer. The results showed that Ch-G intercalation in shale clay minerals was a prerequisite for inhibiting shale hydration swelling.

#### 3.4.3. XPS

The compositions of GO/MMT and Ch-G/MMT hybrids are reflected in Table 1. The nitrogen atom content increased significantly (from 0.44 to 4.89), indicating that the modification of the choline chloride moieties was successful. With the increase in the nitrogen atom content, it is likely that more ionic bonds were formed in the solution.

**Table 1:** Elemental analysis of different inhibitors and MMT hybrids

Sample	O	C	Si	Al	N
Ch-G/MMT	47.65	27.82	13.74	5.88	4.89
GO/MMT	54.34	28.09	11.52	5.58	0.44

#### 3.4.4. SEM

**Fig. 7:**
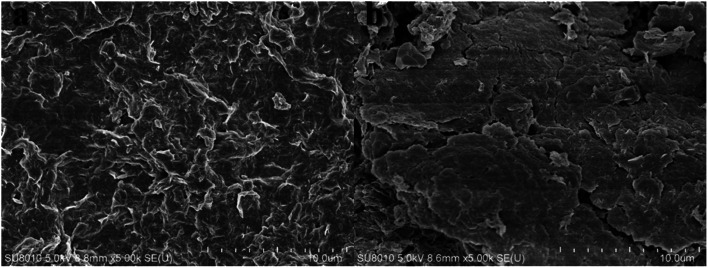
SEM images of (a) pristine MMT and (b) the 0.001 wt% Ch-G/MMT hybrid.

Scanning electron microscopy was used to characterize the morphology of MMT and Ch-G/MMT.^39,40^ SEM images of pristine MMT and the 0.001 wt% Ch-G/MMT hybrid are shown in Fig. 7. There was a big difference between the two SEM images. Ch-G/MMT was only slightly hydrated as compared to the heavily hydrated swollen MMT. The clay particle size after adding Ch-G was larger than that of MMT. The results suggested that Ch-G acted as an inhibitor against the hydration of clay particles.

#### 3.4.5. TEM

**Fig. 8:**
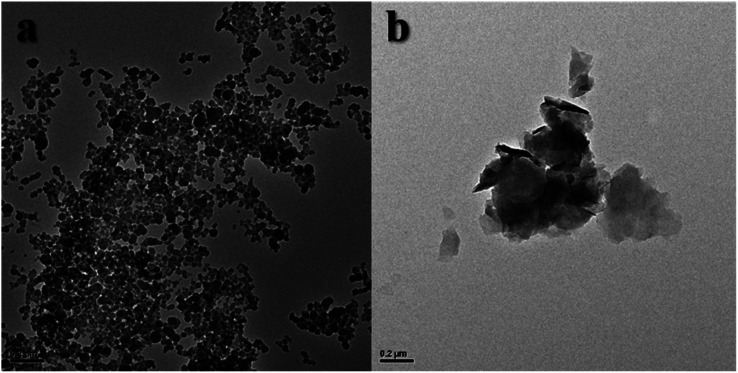
TEM images (a) pristine MMT and (b) the 0.001 wt% Ch-G/MMT hybrid.

Fig. 8 showed TEM images. Due to the hydration of MMT, no clay flakes were observed in Fig. 8(a), and many small nanoflakes were observed at 200 nm. This indicated that the MMT undergoes severe hydration swelling. Larger clay flakes instead of small nanoflakes could be seen, and the MMT layers were stacked on each other. This was due to the inhibition of Ch-G, which inhibited the hydration of clay particles.

#### 3.4.6. AFM

**Fig. 9:**
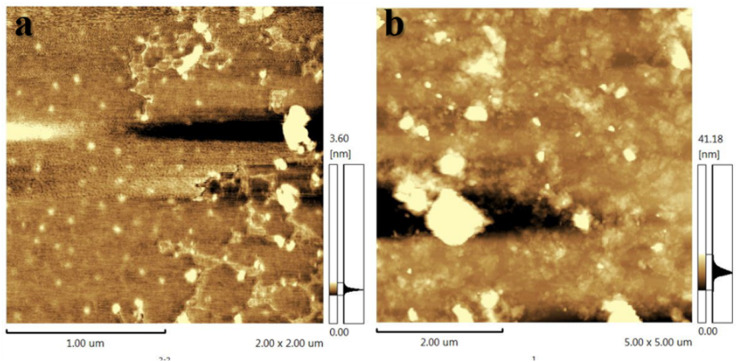
AFM images of (a) pristine MMT and (b) the 0.001 wt% Ch-G/MMT hybrid.

The inhibition mechanism of MMT and Ch-G was also characterized by AFM, and their images were shown in Fig. 9. The MMT was well hydrated and many nanosheets were observed in Fig. 9(a) with a nanosheet height of 3.60 nm. The opposite result was observed in Fig. 9(b). Clay platelets had a height of 41.18 nm after adding 0.001 wt% Ch-G. The results showed that after adding a small amount of Ch-G, the clay particles were not severely hydrated but inhibited.

### 3.5 Probable inhibition mechanism

Ch-G inhibition was investigated and it was found that Ch-G was better than the traditional inhibitor KCl at inhibiting the hydration swelling of clay particles in shale. The sodium ions between the MMT layers acted as easily hydrated cations to form an electric double layer, so that the MMT could form a thick hydration film for hydration and dispersion. However, Ch-G was adsorbed on the MMT surface through electrostatic attraction and hydrogen bonding, neutralized the negative charge on the MMT surface, and compressed the electric double layer. At the same time, due to the intercalation of Ch-G, water molecules were prevented from entering the interlayer of MMT. Therefore, the MMT clay particles agglomerated into large particles, and hydration dispersion was inhibited. The inhibition of Ch-G was beneficial to the stability of the wellbore.

## 4. Conclusions

In this work, we prepared choline chloride-modified graphene (Ch-G) as a shale inhibitor and its inhibition mechanism was investigated. The inhibition of Ch-G was better than that of the traditional inhibitor KCl. Compared with MMT in aqueous solution and KCl solution, the addition of Ch-G achieved the lowest swelling height of 2.10 mm. Rolling recovery tests showed that Ch-G maintained the highest recovery rate of 78.87% after rolling at 120 °C. The inhibition mechanism was discussed further. Hydrogen bonds and ion bands were formed between Ch-G and the MMT surface. With the increase in the nitrogen atom content, the positive ions in the solution increased, which neutralized the negative charge on the surface of MMT and inhibited the expansion of the diffused electric double layer. The intercalation of Ch-G in MMT further prevented water intrusion and optimized the inhibition effect. The results showed that Ch-G could effectively inhibit shale expansion as an environmentally friendly shale inhibitor. On the other hand, the compatibility of Ch-G with other reagents commonly used in drilling fluids needs to be further studied. The next step is in progress.

## Conflicts of interest

There are no conflicts to declare.

## Supplementary Material

## References

[cit1] Kazak E. S., Kazak A. V. (2019). J. Pet. Sci. Eng..

[cit2] Hammad Rasool M., Ahmad M., Ayoub M., Zamir A., Adeem Abbas M. (2022). J. Mol. Liq..

[cit3] Abbas M. A., Zamir A., Elraies K. A., Mahmood S. M., Rasool M. H. (2021). J. Pet. Sci. Eng..

[cit4] An Y., Yu P. (2018). J. Pet. Sci. Eng..

[cit5] Saleh T. A., Rana A., Arfaj M. K. (2020). Environ. Nanotechnol., Monit. Manage..

[cit6] Wang K., Jiang G., Li X., Luckham P. F. (2020). Colloids Surf., A.

[cit7] Huadi F. , Aldea C., Mackereth B. and Mukhlis T., presented in part at the IADC/SPE Asia Pacific Drilling Technology Conference and Exhibition, 2010

[cit8] Suter J. L., Coveney P. V., Anderson R. L., Greenwell H. C., Cliffe S. (2011). Energy Environ. Sci..

[cit9] Davis II N., Tooman C. E. (1989). SPE Drill. Completion.

[cit10] Gholizadeh-Doonechaly N. , Tahmasbi K. and Davani E., presented in part at the SPE International Symposium on Oilfield Chemistry, 2009

[cit11] Guo J., Yan J., Fan W., Zhang H. (2006). J. Pet. Sci. Eng..

[cit12] Aftab A., Ismail A. R., Ibupoto Z. H. (2017). SPE Drill. Completion.

[cit13] Luo Z., Wang L., Yu P., Chen Z. (2017). Appl. Clay Sci..

[cit14] Zhao X., Qiu Z., Sun B., Liu S., Xing X., Wang M. (2019). J. Pet. Sci. Eng..

[cit15] Chen G., Yan J., Lili L., Zhang J., Gu X., Song H. (2017). Appl. Clay Sci..

[cit16] Patel A. D. , presented in part at the SPE International Symposium on Oilfield Chemistry, 2009

[cit17] Xiong K. , Ma P., Yong F., Qian F., Yang R. and Meng Y., presented in part at the IADC/SPE Asia Pacific Drilling Technology Conference and Exhibition, 2012

[cit18] Ma F., Pu X., Wang B., Li J., Cao C. (2017). RSC Adv..

[cit19] Li X., Jiang G., Yang L., Wang K., Shi H., Li G., Wu X. (2019). Energy Fuels.

[cit20] Xu J.-g., Qiu Z., Zhao X., Mou T., Zhong H., Huang W. (2018). RSC Adv..

[cit21] Huang X., Sun J., Jin J., Lv K., Li H., Rong K., Zhang C., Meng X. (2021). J. Mol. Liq..

[cit22] Jia H., Huang P., Wang Q., Han Y., Wang S., Dai J., Song J., Zhang F., Yan H., Lv K. (2020). J. Mol. Liq..

[cit23] Ren Y., Wang H., Ren Z., Zhang Y., Geng Y., Wu L., Pu X. (2019). Appl. Clay Sci..

[cit24] Ma J., Yu P., Xia B., An Y., Wang Z. (2019). ACS Appl. Bio Mater..

[cit25] Xuan Y., Jiang G., Li Y., Yang L., Zhang X. (2015). RSC Adv..

[cit26] Gou S., Yin T., Xia Q., Guo Q. (2015). RSC Adv..

[cit27] Geim A. K., Novoselov K. S. (2007). Nat. Mater..

[cit28] Wu Q., Xu Y., Yao Z., Liu A., Shi G. (2010). ACS Nano.

[cit29] Paul G., Hirani H., Kuila T., Murmu N. C. (2019). Nanoscale.

[cit30] Kosynkin D. V., Ceriotti G., Wilson K. C., Lomeda J. R., Scorsone J. T., Patel A. D., Friedheim J. E., Tour J. M. (2012). ACS Appl. Mater. Interfaces.

[cit31] Ma J., Xu J., Pang S., Zhou W., Xia B., Yuxiu A. (2021). Energy Fuels.

[cit32] Jingyuan M., Boru X., Yuxiu A. (2022). J. Pet. Sci. Eng..

[cit33] Lv K., Huang P., Zhou Z., Wei X., Luo Q., Huang Z., Yan H., Jia H. (2020). Front. Chem..

[cit34] Francisco M., van den Bruinhorst A., Kroon M. C. (2012). Green Chem..

[cit35] Yuxiu A., Guancheng J., Yourong Q., Xianbin H., He S. (2016). J. Nat. Gas Sci. Eng..

[cit36] Che J., Shen L., Xiao Y. (2010). J. Mater. Chem..

[cit37] Yuan W., Liu A., Huang L., Li C., Shi G. (2013). Adv. Mater..

[cit38] Saleh T. A. (2015). Environ. Sci. Pollut. Res..

[cit39] Saleh T. A. (2016). Desalin. Water Treat..

[cit40] Saleh T. A. (2015). J. Water Supply: Res. Technol.--AQUA.

